# Discovery of a highly potent novel rifampicin analog by preparing a hybrid of the precursors of the antibiotic drugs rifampicin and clofazimine

**DOI:** 10.1038/s41598-020-80439-2

**Published:** 2021-01-13

**Authors:** Pasupathy Saravanan, V. N. Azger Dusthackeer, R. S. Rajmani, B. Mahizhaveni, Christy R. Nirmal, Sam Ebenezer Rajadas, Neerupma Bhardwaj, C. Ponnuraja, Adhin Bhaskar, A. K. Hemanthkumar, Geetha Ramachandran, Srikanth P. Tripathy

**Affiliations:** 1 ICMR-National Institute for Research in Tuberculosis, Chennai, India; 2 Centre for Infectious Disease and Research, Indian Institute of Science, Bangalore, India

**Keywords:** Microbiology, Chemistry

## Abstract

Tuberculosis (TB) is an infectious disease caused by the bacillus *Mycobacterium tuberculosis* (Mtb). The present work reports the design and synthesis of a hybrid of the precursors of rifampicin and clofazimine, which led to the discovery of a novel Rifaphenazine (RPZ) molecule with potent anti-TB activity. In addition, the efficacy of RPZ was evaluated in-vitro using the reference strain Mtb H37Rv. Herein, 2,3 diamino phenazine, a precursor of an anti-TB drug clofazimine, was tethered to the rifampicin core. This 2,3 diamino phenazine did not have an inherent anti-TB activity even at a concentration of up to 2 µg/mL, while rifampicin did not exhibit any activity against Mtb at a concentration of 0.1 µg/mL. However, the synthesized novel Rifaphenzine (RPZ) inhibited 78% of the Mtb colonies at a drug concentration of 0.1 µg/mL, while 93% of the bacterial colonies were killed at 0.5 µg/mL of the drug. Furthermore, the Minimum Inhibitory Concentration (MIC) value for RPZ was 1 µg/mL. Time-kill studies revealed that all bacterial colonies were killed within a period of 24 h. The synthesized novel molecule was characterized using high-resolution mass spectroscopy and NMR spectroscopy. Cytotoxicity studies (IC_50_) were performed on human monocytic cell line THP-1, and the determined IC50 value was 96 µg/mL, which is non-cytotoxic.

## Introduction

Tuberculosis (TB), a disease caused by an infective agent named *Mycobacterium tuberculosis* (Mtb), is one of the top ten causes of death in humans, ranking above HIV/AIDS. According to a recent report by World Health Organization (WHO), an estimated 1.7 billion people are infected with *M. tuberculosis* worldwide, among which around 5–10% advance into the TB disease during their lifetime^1^. The year 2018 alone registered ten million new TB cases and 1.5 million deaths, which included 251,000 TB deaths among HIV-positive individuals. The drug-susceptible TB patients are recommended rifampicin, isoniazid, ethambutol, and pyrazinamide for a period of six months^2^, and the treatment success rate for these patients is only 85%. Rifamycins are effective against TB-related mycobacterial infections, although their prolonged usage may cause rifampicin-resistance in certain strains of *Mycobacterium tuberculosis* (RR-TB). The individuals infected with RR-TB or multidrug-resistant TB (MDR-TB) require treatment with further expensive and toxic drugs for a longer period, which leads to a complex treatment procedure. The success rate of MDR-TB globally is quite low, around 56% only, which is mainly due to the non-existence of highly effective drugs in the market. Therefore, it is crucial to develop novel anti-TB drugs with higher potency, minimum side-effects, enhanced pharmacokinetic properties, and overall shorter treatment duration. The development of highly-effective antibiotics by introducing new chemical groups into the core of the chemical structures of the antibiotic to increase its anti-bacterial activity continues to be an interesting challenge for the scientific community^3^. When two or more different pharmacophores of well-recognized drugs known to possess desired anti-bacterial properties are tethered using synthetic methods, antibiotic hybrids are generated^4^. TenNor Therapeutics Ltd completed the phase II studies concerning the anti-bacterial activity of Rifampicin-Ciprofloxacin (TNP-2092) multi-target drug conjugate through the inhibition of important drug targets, such as RNA polymerase, topoisomerase IV, and DNA gyrase^5–7^. Other commercially-available Rifampicin analogs, such as Rifabutin, are prescribed for HIV-infected patients with TB, while Rifapentene is prescribed for a shorter regimen and as a preventive therapy. Since there is a lack of highly-effective Rifampicin analogs, researchers are now studying high-dose rifampicin for drug-susceptible TB patients, and these studies are currently in Phase II trials^8,9^. Several other promising anti-TB agents with different target sites have been reported recently in the literature^10–16^. Although, to the best of our knowledge, there is no report on a Rifampicin-Phenazine hybrid drug and its anti-TB activity in the literature so far.

## Results

**Figure 1:**
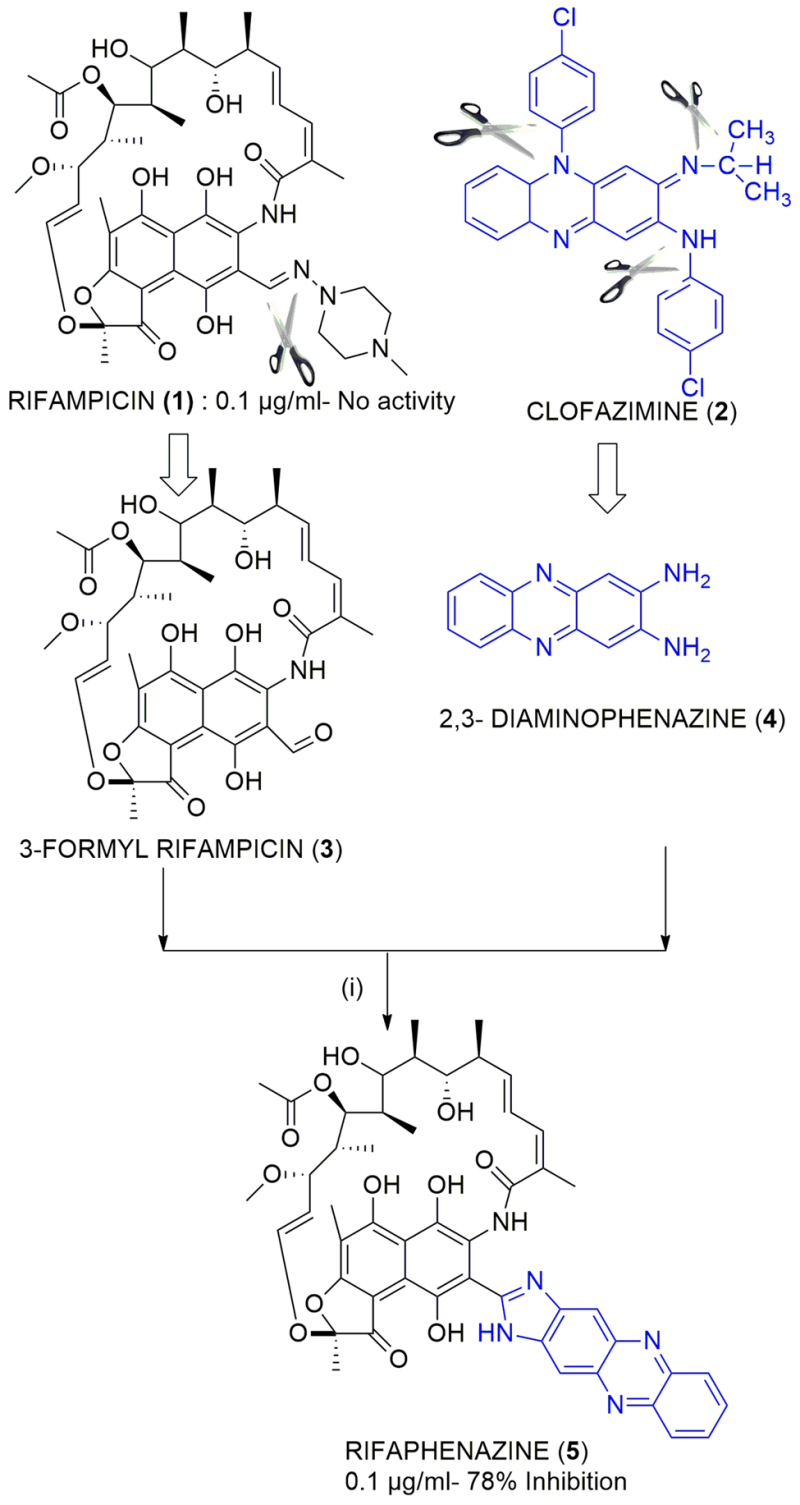
Synthesis of the hybrid molecule RPZ (**5**). Reagents and conditions: (i) Glacial acetic acid, MeOH, 45 ℃, 2 h.

With the aim of developing better analogs of rifampicin, an analog of rifampicin named Rifaphenazine (RPZ) was synthesized in the present study by preparing a hybrid of the precursors of the approved drugs rifampicin (RIF) and clofazimine (CFZ). This analog was synthesized through a rapid single-step procedure illustrated in Fig. 1. Briefly, formyl rifampicin **3** was allowed to react with 2,3 diaminophenazine **4** in the presence of glacial acetic acid, producing Rifaphenazine (RPZ) **5** with a moderate yield of 53%. The structure of the synthesized analogs was characterized using high-resolution mass spectrometry, 1H NMR, 13C NMR, and 2D NMR techniques such as heteronuclear multiple bond correlation (HMBC) and heteronuclear single quantum correlation (HSQC) spectroscopy. The calculated molecular weight of the hybrid drug RPZ (C_50_H_53_N_5_O_12_) was 915.3700, while the experimentally observed molecular weight in high-resolution mass spectroscopy (HRMS) was *m/z* 916.3951(M + 1)+.

**Figure 2:**
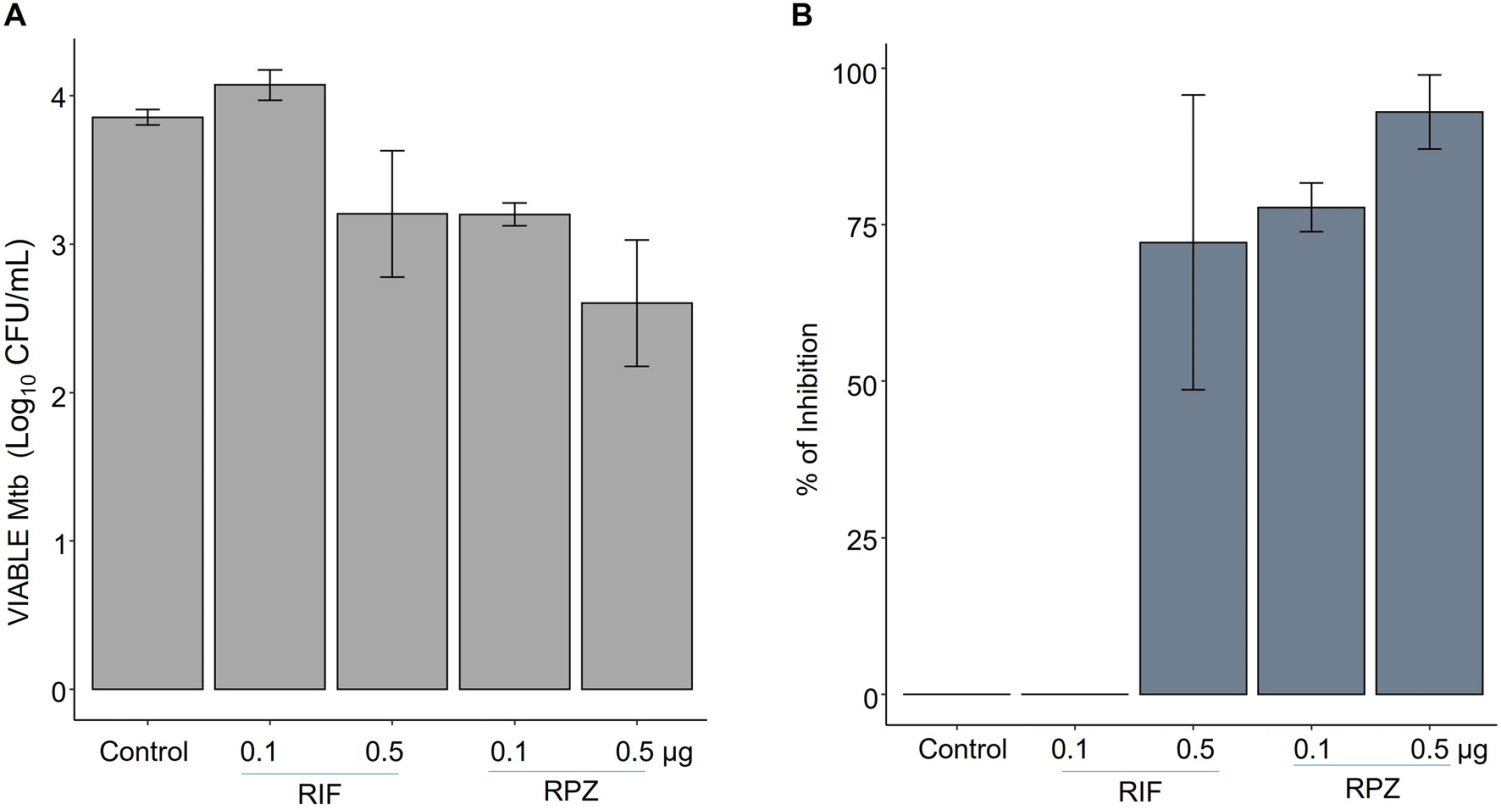
The in-vitro anti-TB activity of drugs against Mtb strain H37Rv*.* (**A**) The remaining Mtb (CFU) left over after treatment with RPZ and control drug RIF are shown in this graph. (**B**) The plot shows % of Inhibition of Mtb H37Rv by RPZ. Results are mean of the vertical bars represent ± SD of two experiments. The difference in the number of CFU between RIF 0.5 VS RPZ 0.5, RIF 0.5 VS RPZ 0.1 and RPZ 0.5 VS RPZ 0.1 were statistically significant. (P < 0.001). The statistical analyses were performed using R software version 3.6.1 (R Core Team (2019). R: A language and environment for statistical computing. R Foundation for Statistical Computing, Vienna, Austria. URL https://www.R-project.org/).

The antituberculosis activity of Rifaphenazine (RPZ) against the *M. tuberculosis* H37Rv strain was evaluated under in-vitro conditions using the broth microdilution method followed by spotting the drug-treated culture onto Middlebrook 7H11 agar supplemented with OADC (oleic acid, bovine serum albumin, dextrose, and catalase). The drug candidate RPZ at the drug concentration of 0.5 µg/mL exhibited 1.3log_10_ reduction in the bacterial load compared to the control (without drug), while the positive control RIF exhibited 0.7log_10_ difference only. At a lower drug concentration of 0.1 µg/mL, RPZ exhibited a difference of 0.7 log_10_ compared to the control (without drug), while RIF at the same drug concentration did not exhibit any activity at all. Figure 2 depicts the graphical representation of drug vs. viable Mtb log_10_ CFU/mL.

The drug efficacy, i.e., the concentration of the drug vs. the % inhibition, is presented in Fig. 2. A 78% Mtb inhibition was observed for RPZ at 0.1 µg/mL, while the positive control drug RIF did not exhibit any inhibition at the same concentration. Similarly, at a higher concentration of 0.5 µg/mL, RPZ exhibited Mtb inhibition of 93%, while RIF exhibited only 72% inhibition.

### Minimum inhibitory concentration (MIC) study

The results from the Minimum Inhibitory Concentration (MIC) experiments are presented in Table 1. The MIC study^17^ was conducted against the Mtb H37Rv strain using the broth microdilution method. It was observed that the drug RPZ exhibited MIC at 0.5 µg/mL, while RIF exhibited MIC at 1 µg/mL. The reproducibility of these results was confirmed through another MIC study using the Mtb H37Rv strain that was passaged freshly and isolated from the guinea pig, and it was observed that both RPZ and RIF exhibited MIC at 1 µg/mL. Moreover, the clofazimine precursor Phenazine (4) did not exhibit any activity up to the concentration of 2 µg/mL.

**Table 1:** Minimum inhibitory concentration (MIC) of drugs and the results of cytotoxicity studies.

Drugs	Mtb H37Rv strain		Mtb H37Rv strain (freshly passaged from guinea pig)		Cytotoxicity^b^
	1 µg/mL	0.5 µg/mL	1 µg/mL	0.5 µg/mL	
RIF	(**Inhibited**)	Scanty^a^	(**Inhibited**)	Scanty^a^	87 µg/mL
Phenazine(4)	(No Inhibition)	(No Inhibition)	(No Inhibition)	(No Inhibition)	–
RPZ (5)	(**Inhibited**)	(**Inhibited**)	(**Inhibited**)	Scanty^a^	96 µg/mL

^a^Able to see the growth of very few countable colonies.

^b^(IC_50_ on human monocytic cell line THP-1).

### Time-kill assay

**Figure 3:**
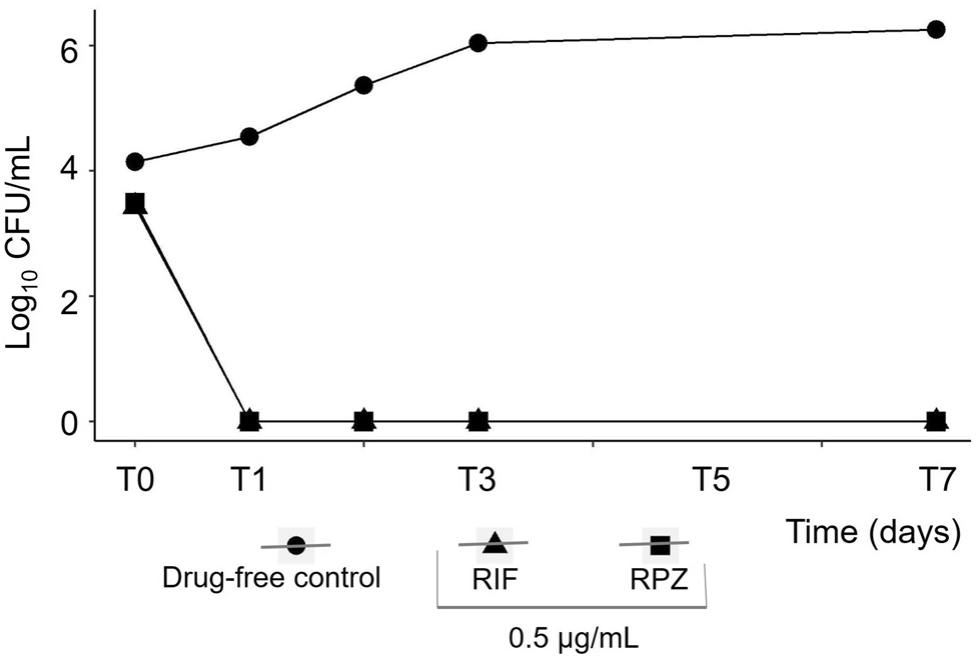
Time-kill curves of the Mtb H37Rv strain when exposed to RPZ and RIF for seven days.

Exposure of Mtb H37Rv to RPZ at a concentration of 0.5 μg/mL was observed to reduce the Mtb population by 3Log_10_ within 24 h of drug exposure. Even after seven days of exposure, no emergence of the Mtb surviving population was observed, indicating the drug’s Mtb-sterilizing property and stability. The time-dependent bactericidal property of RPZ is similar to that of the rifampicin drug. (Fig. 3).

### Cytotoxicity assay

The cytotoxicity of RPZ was assessed ex-vivo using human monocytic cell line THP-1, and its IC_50_ was estimated. RPZ presented an IC_50_ value of 96 µg/mL, while RIF presented an IC_50_ of 87 µg/mL (Table 1). With IC_50_ > 90 μg/mL, RPZ could be classified as a non-cytotoxic compound.

## Discussion

**Figure 4:**
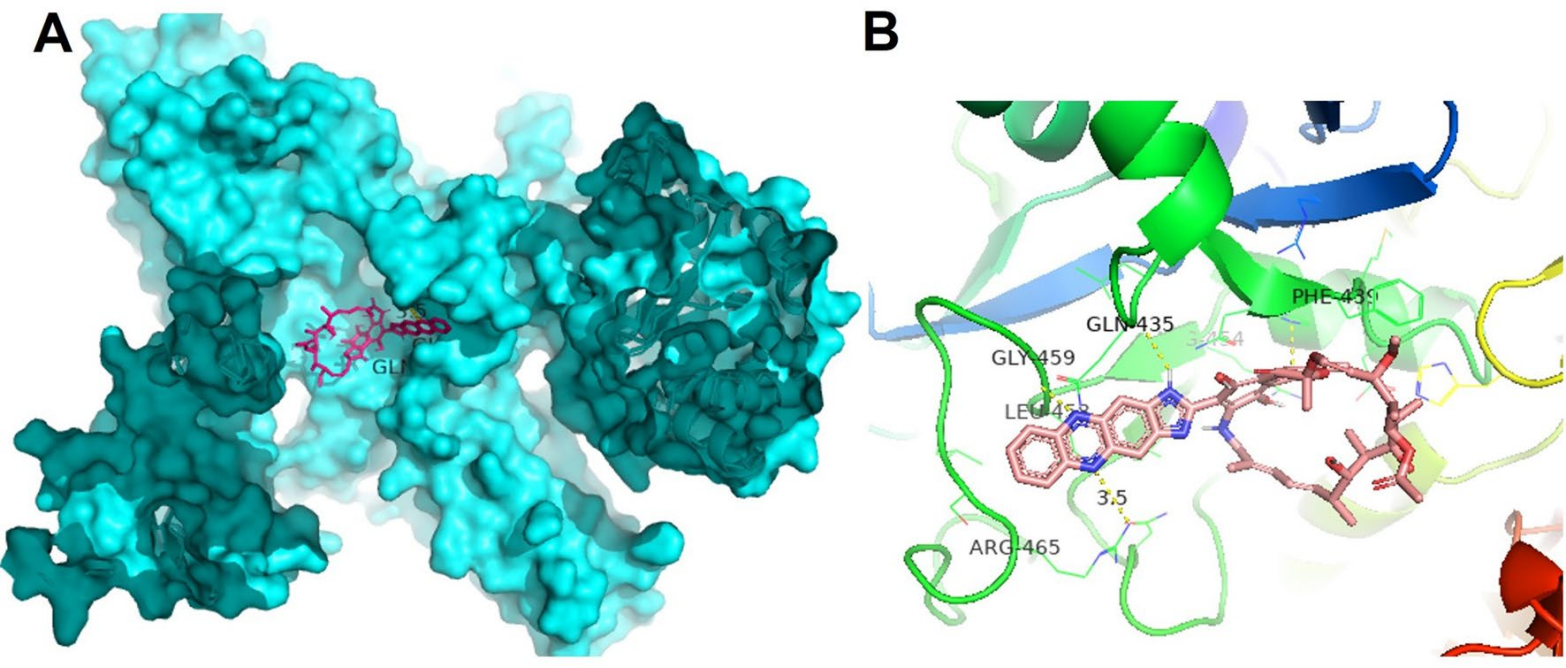
(**A**) Binding of RPZ at the active site of Mtb RNA polymerase. (**B**) The yellow lines depict the hydrogen bond interactions of the residues in the β-chain of the RNA polymerase with the inhibitor RPZ. The figure was visualized using the PyMOL molecular graphics system (Version 2.4.0, Schrödinger, LLC, https://www.pymol.org).

The present study reports the successful synthesis of the novel Rifaphenazine (RPZ) molecule and proposes it as a potential and safe anti-TB drug based on the in-vitro analysis. The RPZ molecule was able to arrest the growth of MTB even at a lower drug concentration. The rifampicin (RIF) drug generally acts by inhibiting the bacterial DNA-dependent RNA polymerase specifically, which is achieved through the formation of a stable complex of the drug with the ß-subunit of the RNA polymerase^18^. Since RPZ is structurally similar to RIF, it was expected that RPZ may also bind with RNA polymerase and inhibit the growth of Mtb. Besides, our in-silico molecular docking simulation study demonstrates that RPZ binds with the active site of Mtb RNA polymerase with a binding affinity of − 8.2 kcal/mol (Fig. 4).

Rifampicin is an important first-line anti-TB drug, while drugs such as clofazimine (CFZ) are used in second-line anti-TB treatment. However, CFZ has severe side effects, including hyper pigmented patches appearing on the skin^19^. The two chlorobenzene moieties attached to the phenazine core of CFZ might be the reason for these side effects. If chlorobenzene and dimethyl methane are removed from the structure of the CFZ drug using the retrosynthetic approach, it produces 2,3-diamino phenazine at the main core of the CFZ drug. Therefore, the same 2,3-diamino phenazine was obtained from a commercial source, and its anti-TB activity was evaluated. However, the results revealed that the drug precursor (2,3-diamino phenazine) had not shown any anti-TB activity. Subsequently, it was hypothesized that, in order to recover the anti-TB activity that was lost due to the altered chemical properties of 2,3-diaminophenazine during the process of making the drug more effective and less toxic, it is necessary to form a hybrid of this drug precursor with another active pharmacophore, i.e., Rifampicin core. The reaction mechanism of the synthesis of Rifaphenazine **5** involved rapid condensation of the formyl group in rifampicin with an amino group in phenazine in the presence of glacial acetic acid as catalyst, followed by cyclization. Interestingly, the synthesized hybrid was observed to be more potent than the Rifampicin drug against the Mtb H37Rv strain. Furthermore, due to the aromatic nature of the attached phenazine ring, the synthesized analog (RPZ) was relatively more stable than RIF and could, therefore, be stored at ambient temperatures. The 1H NMR spectrum of RPZ revealed six aromatic protons of the phenazine ring that resonated between δ7.63 ppm and δ8.26 ppm, while the amide N–H proton appeared at δ11.79. The HMBC spectrum revealed that the N–H proton correlated with the naphthyl carbon atoms in the RIF core, while the 1H-13C of HSQC spectrum revealed that the six aromatic protons of the phenazine ring correlated with the carbon atom that appeared between δ106 and δ129.

In conclusion, these data suggested that Rifaphenazine (RPZ) is a promising pre-clinical candidate for the treatment of drug-susceptible tuberculosis (DS-TB). The results of the in-vitro analysis suggest that the efficacy of Mtb inhibition demonstrated by RPZ at a lower drug concentration has a prospect of shortening the TB treatment duration. The significance of the present work is that, even at a one-tenth concentration of rifampicin MIC, the drug candidate Rifaphenazine (RPZ) was able to kill 78% of Mtb, while rifampicin could kill zero percent Mtb at the same concentration. Furthermore, the cytotoxicity studies revealed that RPZ was not cytotoxic to the human monocytic cell line THP-1. Future research should endeavor to perform pre-clinical studies with different sets of bacterial strains and Mtb clinical strains to provide a strong lead to clinical studies.

## Materials and methods

### Chemistry

The synthesized compounds were characterized through 1H-NMR and 13C NMR using Bruker Avance 500 spectrometer and CDCl_3_ as the solvent. High-resolution mass spectra (HRMS) were obtained by using Agilent Q-TOF-Mass Spectrometer in the ESI positive mode, and the values were expressed in *m/z*. The synthesized drug analog was purified through column chromatography using glass columns packed with silica gel (100–200 mesh). Thin Layer Chromatography (TLC) was performed using Merck aluminium-backed TLC Silica gel plate (60 F254). The chemicals and solvents used in the analyses were obtained from commercial sources and used as received.

### General procedure for the synthesis of the RIF analog

#### Synthesis of Rifaphenazine 5

Formyl rifampicin **3** (1 equivalent) and 2,3 diamino phenazine **4** (1.2 equivalent) were added to a suspension of glacial acetic acid (100 µL) in MeOH (15 mL) at 45 ℃, followed by stirring for 2 h to allow the reaction to occur. After the completion of the reaction, the solvent was removed under vacuum and the solid product was extracted with Dichloromethane (DCM)/Water (3 × 10 mL). The collective DCM extracts were dried with sodium sulphate (Na_2_SO_4_) and then concentrated to obtain the final product **5** in solid form. The produced compound was purified through column chromatography using the mixture of dichloromethane and methanol as solvent. TLC (DCM:MeOH, 95:5 v/v): R_f_ = 0.75; ^1^H NMR (500 MHz, CDCl_3_): δ 15.25 (br s, 1H), 14.09 (s, 1H), 13.41–13.33 (m, 1H), 11.79 (s, 1H), 8.26 (s, 1H), 8.12 (d, J = 10 Hz, 1H), 7.89 (d, J = 10 Hz, 2H), 7.70 (t, J = 15 Hz, 1H), 7.64 (t, J = 15 Hz, 1H), 6.58–6.49 (m, 1H), 6.42–6.37 (m, 1H), 6.17 (d, J = 15 Hz, 1H), 5.87 (dd, J = 5 Hz, 1H), 5.15–5.10 (m, 1H), 4.85 (d, J = 10 Hz, 1H), 3.52–3.44 (m, 2H), 3.26 (br s, 1H), 3.20 (d, J = 10 Hz, 1H), 3.05–3.02 (m, 3H), 2.76 (d, J = 10 Hz, 1H), 2.31 (s, 3H), 2.26 (s, 1H), 2.19 (s, 3H), 2.08–2.05 (m, 2H), 2.01–1.07 (m, 6H), 1.30–1.22 (m, 4H), 1.11–1.09 (m, 1H), 1.03 (d, J = 10 Hz, 1H), 0.71–0.60 (m, 3H), 0.16 (d, J = 10 Hz, 3H), -0.34 to -0.38 (m, 6H); ^13^C NMR (125 MHz, CDCl_3_): δ 171.96, 156.49, 150.00, 142.51, 142.47, 142.21, 140.39, 139.84, 135.54, 129.97, 129.58, 129.29, 128.85, 123.20, 119.30, 118.31, 115.78, 114.55, 109.20, 106.23, 74.00, 70.46, 60.43, 57.25, 39.41, 37.93, 37.29, 32.83, 29.70, 21.45, 20.91, 20.68, 16.29, 10.51, 8.85, 8.24, 7.57; HRMS (*m/z*): [M]^+^calcd. for C_50_H_53_N_5_O_12_, 915.3700; experimentally observed, 916.3951(M + 1)+.

### The antituberculosis activity of RPZ against Mtb H37Rv

The Minimum Inhibitory Concentration (MIC) study was performed against the *M. tuberculosis* H37Rv strains with different susceptibilities (standard H37Rv strain and the same strain passaged recently and isolated from guinea pig) using the broth microdilution method^17^, and the results are presented in Table 1. Briefly, the Mtb culture in Middlebrook 7H9 broth at the log-phase of growth was adjusted to a McFarland standard turbidity of 0.5 and allowed to settle for fifteen minutes. Subsequently, this culture was added to the wells of a 96-well microtiter plate to reach the final drug concentrations ranging from 0.1 to 2.0 µg/mL. Among the wells, two wells represented drug-free (inoculum-only) controls. After 7 days of incubation, both drug-free and drug-treated wells were examined under an inverted phase-contrast microscope to observe the characteristic serpentine cord formation of Mtb. The lowest drug concentration that inhibited the microscopically-visible growth of Mtb after seven days was referred to as the Minimum Inhibitory Concentration (MIC) of the drug. In order to ascertain the bactericidal property of RPZ, the Mtb Colony-forming units (CFU) were enumerated by spotting a specified volume of serially-diluted aliquots drawn from the corresponding wells onto Middlebrook 7H11 agar supplemented with OADC [oleic acid, bovine serum albumin, dextrose, and catalase (Becton–Dickinson)]. The assay was performed in triplicate. The plates were incubated at 37 °C and the colonies were counted after 21 days of incubation.

### Time-kill assay

In order to further evaluate the efficacy of RPZ, a time-kill assay was performed against the *M. tuberculosis* H37Rv laboratory strain using the method described by Demitto et al.^14^. In brief, *M. tuberculosis* H37Rv grown in the Middlebrook 7H9 medium (7H9) (Difco Laboratories, Detroit, MI, USA) supplemented with OADC, 0.2% glycerol, and 0.025% Tween 80 was diluted to a McFarland standard turbidity of 1, to yield a cell population of 3 × 10^8^ colony-forming units [CFU]/mL, which was subsequently diluted in 7H9 to obtain a final concentration of 10^4^ CFU/mL. RPZ at the final concentration of 0.5 μg/mL was added to this cell suspension in duplicates in individual tubes that were protected from light. A similar concentration of rifampicin (RIF) was used as a positive control, besides the unexposed Mtb culture control. Both drug-treated and un-treated Mtb cell suspensions were incubated at 37 ℃ in an orbital shaker. Post-drug exposure, aliquots were retrieved every 24 h for 7 days. Each of these aliquots was serially-diluted, and then 5 μL of each dilution was spot-inoculated on OADC-supplemented Middlebrook 7H11 agar, followed by incubation at 37 ℃. The CFUs were determined as the number of colonies corresponding to each dilution. The assay was performed in duplicate, while the spotting from each dilution was performed in triplicate. The average of the CFU values for these triplicates was used as the final CFU.

### Cytotoxicity: drug sensitivity assays (IC_50_)

The cytotoxicity of RPZ was assessed using the human monocytic cell line THP-1. The THP1 cell suspension was cultured in RPMI-1640 medium supplemented with 10% fetal bovine serum (FBS) and maintained at 37 ℃ in 5% CO_2_ atmosphere. The monocytes were differentiated by exposing the cells to 20 ng/mL of phorbol 12-myristate 13-acetate (PMA) for 24 h. The cells were allowed to rest for 2 days following the chemical differentiation to ensure that they attain a resting phenotype prior to the RPZ exposure. Briefly, the stock drug solutions with a concentration of 10 mg/mL were prepared in 100% dimethylsulphoxide (DMSO) and were further diluted to the required working solution concentration using RPMI-1640 complete media. The cytotoxicity assays were performed in 96-well microtiter plates, with each well receiving 100 μL of the culture medium and 4 × 10^4^ cells/well. The highest concentration of the test compounds was 200 μg/mL. Each drug was tested in duplicate. The plates were examined after 72 h of incubation, and then 10 μL Alamar Blue (12.5 mg resazurin dissolved in 100 mL distilled water) was added to each well, followed by another 2 h of incubation. Subsequently, the microplate reader (Spectramax) was used to measure the optical density at an excitation wavelength of 536 nm and an emission wavelength of 588 nm, and the IC_50_ values were calculated (Supplementary Information).

### Statistical analysis

The statistical analyses were performed using R software version 3.6.1 (R Core Team (2019). R: A language and environment for statistical computing. R Foundation for Statistical Computing, Vienna, Austria. URL https://www.R-project.org/) Pearson’s Chi-squared test was used to compare the proportions of bacteria killed in the presence of each drug. Pairwise comparison of the proportions was performed at a 0.017 significance level. P < 0.05 was considered significant.

### Molecular docking studies

Molecular docking was performed using the Autodock 4.2^®^ program^20^. The complex data files of the Mtb RNAP were obtained from the Protein Data Bank (PDB-5UHC)^21^. The proteins were prepared by removing all the water molecules and ligands using the Autodock Tools program version 1.5.6. The chemical structures of the drug were constructed using Chem3D. The energy minimization was conducted using the MM2 forcefield. A 120 × 120 × 120 and 3.75 Å grid box was set around the active sites for ligand interaction. The rigid grid box was prepared using Auto grid. The best docking conformation was obtained by using AutoDock with the Lamarckian Genetic Algorithm. The remaining docking parameters were set to default values. The binding pose presenting the best binding affinity was visualized using Pymol (Version 2.4.0, Schrödinger, LLC, https://www.pymol.org). Figure 4 depicts the interaction between the inhibitor RPZ and a receptor Mtb RNAP, presenting the H-bonds formed by the inhibitor with the residues present in the active site of the receptor and the non-covalent interactions (− 8.2 kcal/mol) contributing to the Mtb inhibition.

## Supplementary Information

Supplementary Information.
