# Supplementary material for: Discovery of a highly potent novel rifampicin analog by preparing a hybrid of the precursors of the antibiotic drugs rifampicin and clofazimine
Source: Sci Rep. 2021 Jan 13;11:1029. doi: 10.1038/s41598-020-80439-2 (PMC7806721; doi:10.1038/s41598-020-80439-2)
Supplement: Supplementary file 1 — Supplementary Information. [file 41598_2020_80439_MOESM1_ESM.pdf]

Supplementary Information for

Discovery of a Highly Potent Novel Rifampicin analog by preparing a Hybrid of the Precursors of the antibiotic drugs Rifampicin and Clofazimine

Pasupathy Saravanan [a,1], V.N. Azger Dusthackeer [a], R.S. Rajmani [b], B. Mahizhaveni [a], Christy R. Nirmal [a], Sam Ebenezer Rajadas [a], Neerupma Bharadwaj [b], C. Ponnuraja [a], Adhin Bhaskar [a], A.K. Hemanthkumar [a], Geetha Ramachandran [a], and Srikanth P. Tripathy [a,1].

[1] To whom correspondence may be addressed.
Email:saran2k5@gmail.com or srikanthtripaty@gmail.com

This supplementary file includes:

Figures S1 to S5

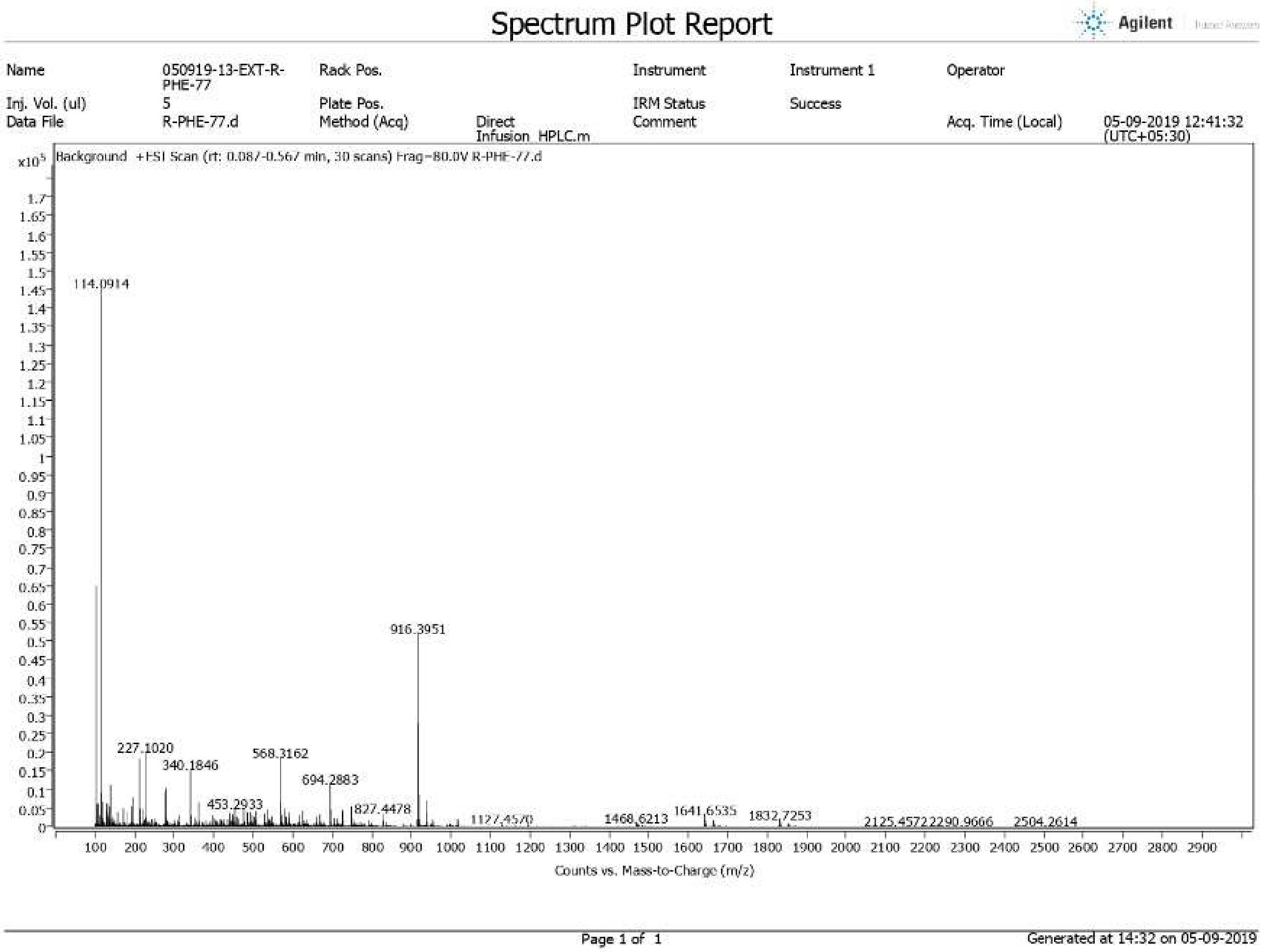

Fig. S1. HRMS spectrum of RPZ (**5**)

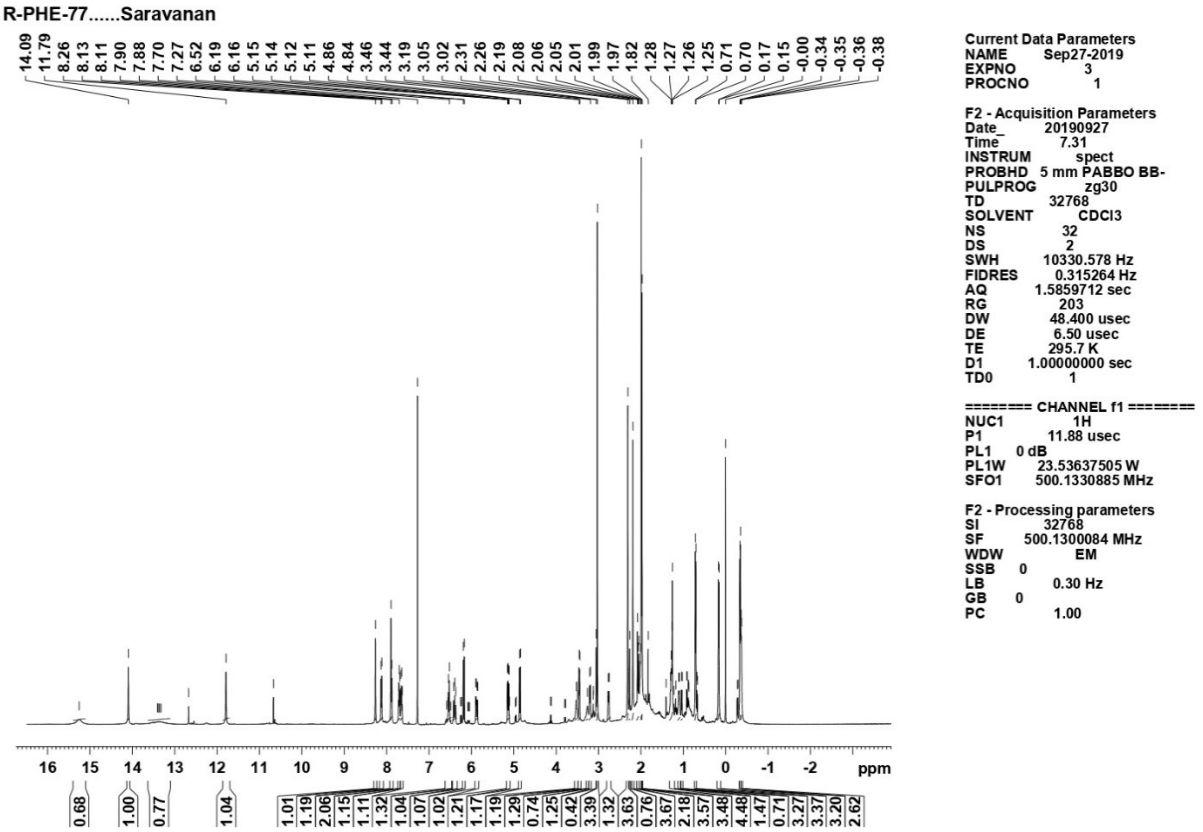

Fig. S2. ^{1}H NMR Spectrum of RPZ (**5**)

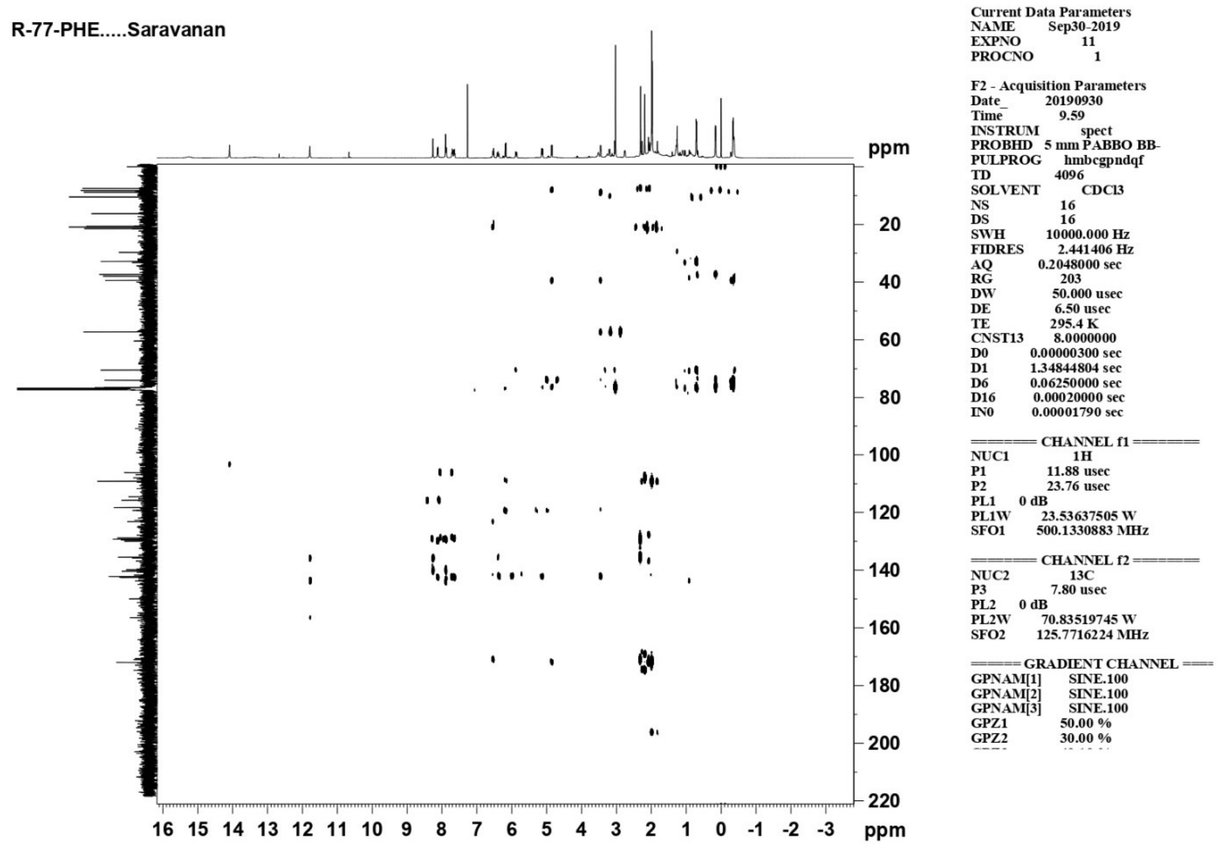

Fig. S3. HMBC Spectrum of RPZ (**5**)

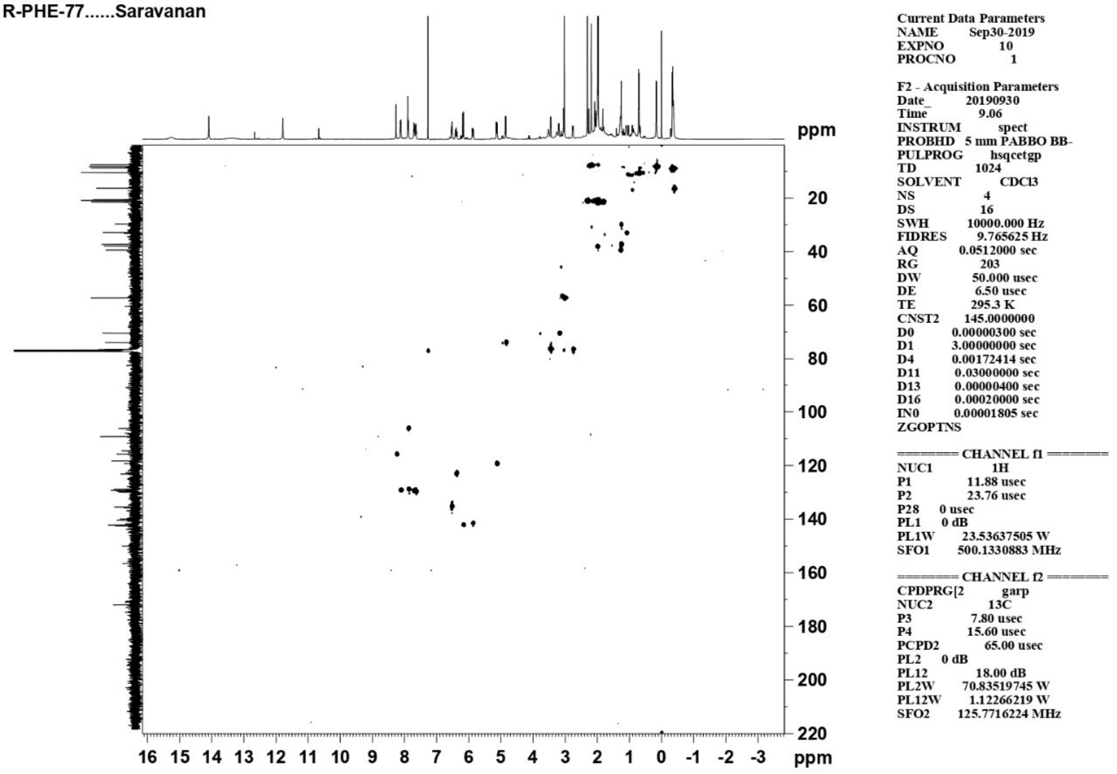

Fig. S4. HSQC Spectrum of RPZ (**5**)

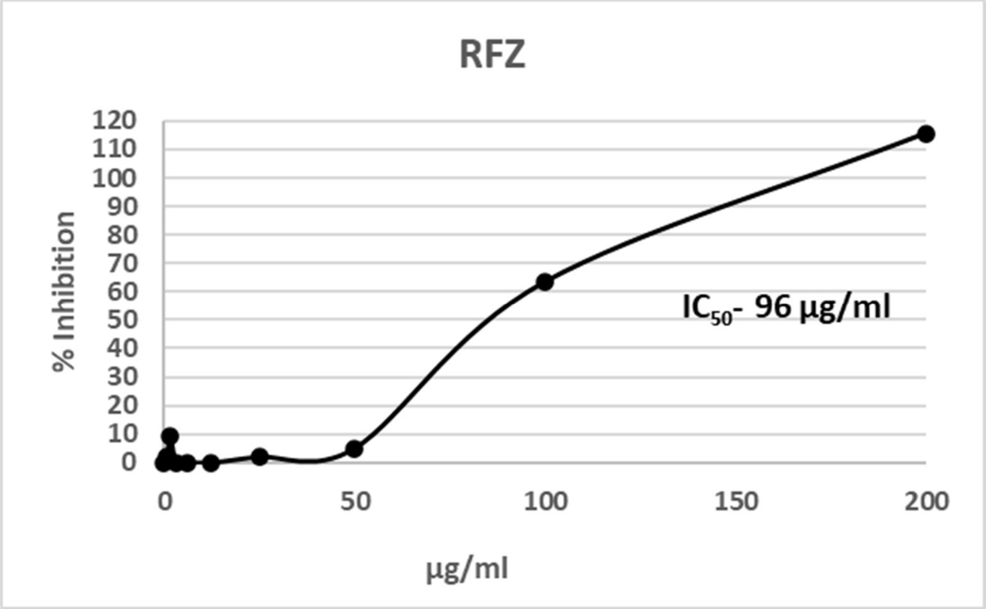

Fig. **S5**. Cytotoxicity (IC_{50}) study of RPZ on human monocytic cell line THP-1
